# Infection Rates and Risk Factors for Infection Among Health Workers During Ebola and Marburg Virus Outbreaks: A Systematic Review

**DOI:** 10.1093/infdis/jiy435

**Published:** 2018-09-07

**Authors:** Saranya A Selvaraj, Karen E Lee, Mason Harrell, Ivan Ivanov, Benedetta Allegranzi

**Affiliations:** 1 University of Maryland Medical Center, Baltimore; 2 University of Dundee School of Nursing and Health Sciences, United Kingdom; 3 Harvard School of Public Health, Boston, Massachusetts; 4 Department of Public Health Environmental and Social Determinants of Health; 5 Department of Service Delivery and Safety, World Health Organization, Geneva, Switzerland

**Keywords:** Ebola virus disease, Marburg virus disease, infection prevention and control, healthcare workers, occupational health

## Abstract

**Background:**

Infection in health workers (HWs) has characterized outbreaks of Ebola virus disease (EVD) and Marburg virus disease (MVD). We conducted a systematic review to investigate infection and mortality rates and common exposure risks in HWs in EVD and MVD outbreaks.

**Methods:**

We searched the EMBASE and PubMed databases to identify articles posted before 27 December 2017, with no language restrictions. Data on the number, frequency, and mortality of HW infection and exposure risks were extracted.

**Results:**

Ninety-four articles related to 22 outbreaks were included. HW infections composed 2%–100% of cases in EVD and 5%–50% of cases in MVD outbreaks. Among exposed HWs, 0.6%–92% developed EVD, and 1%–10% developed MVD. HW infection rates were consistent through outbreaks. The most common exposure risk situations were inadequate personal protective equipment and exposure to patients with unrecognized EVD/MVD. Similar risks were reported in past EVD/MVD outbreaks and in the recent outbreak in West Africa.

**Conclusions:**

Many outbreaks reported high proportions of infected HWs. Similar HW infection rates and exposure risk factors in both past and recent EVD and MVD outbreaks emphasize the need to improve the implementation of appropriate infection control measures consistently across all healthcare settings.

Ebola and Marburg viruses are members of the Filoviridae (filovirus) family and have an extremely high virulence and mortality rate, but no therapeutic treatments are currently available. Following infection from an animal reservoir, human-to-human transmission occurs through direct or indirect contact with blood or body fluids of a person who is infected with or has died from Ebola virus disease (EVD) due to one of the 4 species of *Ebolavirus* pathogenic in humans or from Marburg virus disease (MVD). The recent 2013–2016 EVD outbreak in West Africa, particularly in Guinea, Liberia, and Sierra Leone, was of an unprecedented dimension and severity, leading to 28616 EVD cases and 11310 deaths [1].

Since the first reported outbreaks of MVD, in 1967 [2–4], and EVD, in 1976 [5, 6], health workers (HWs) have been recognized as having an increased risk of infection, owing to their occupational exposure to blood and body fluids, particularly in the absence of appropriate infection prevention and control (IPC) and occupational health and safety measures. In developing countries, HW infection undermines fragile health systems by stretching already thin workforces. Outbreaks of deadly infection among HWs are considered red flags that should trigger suspicion for EVD or MVD and often result in nosocomial spread between staff and patients and then spread back into the community [7–12]. In the 2013–2016 West Africa EVD outbreak, the World Health Organization (WHO) published an interim report indicating a huge impact on HWs, with 861 (3.9%) confirmed or probable cases between 1 January 2014 and 8 April 2015 for Guinea, Liberia, and Sierra Leone combined [13].

Measures to contain outbreaks rely on rapid detection and isolation of cases, contact tracing, IPC in the community and healthcare facilities, and avoidance of funeral practices involving contact with the deceased. International guidelines have been available since 1974 [14–16], but their implementation was initially difficult in the 2013–2016 West Africa EVD outbreak because of the high number of cases and many gaps in infrastructure and supplies in the already challenged health systems of affected countries. We conducted a systematic review of the literature to identify and compare EVD and MVD infection rates among HWs to those of the general population. We also aimed to identify the most affected HW occupations and the most frequent exposure risk situations.

## METHODS

### Search Strategy and Selection Criteria

We identified studies by searching the EMBASE and PubMed databases for articles posted before 27 December 2017, with no time or language restrictions (see Supplementary Tables 1 and 2 for full search terms). HWs were defined as any person at risk for occupational exposure to EVD or MVD, ranging from HWs normally providing patient care, such as nurses, physicians, or traditional healers, to other workers who may have been exposed through their regular occupational duties or through being exceptionally asked to serve in a healthcare setting. Additional articles were identified by searching reference lists by hand from retrieved publications and by reviewing the WHO archives.

After excluding duplicate references, 2 independent reviewers screened the titles and abstracts of retrieved references. Potentially relevant articles were retrieved for full-text review and assessed for study eligibility, again by 2 independent reviewers. Interreviewer disagreement was resolved by consensus or, if consensus could not be reached, by a third reviewer. Inclusion criteria were any mention of EVD/MVD in HWs that was accompanied by epidemiological data related to infection in HWs and/or qualitative descriptions of exposure risk situations and infection prevention practices. We also included published personal accounts, interviews, situation/field reports, and news items. Exclusion criteria were conference abstracts, reviews, and papers not containing primary data related to the research questions or not including any HW infections.

When available, data extraction included type of study or report; type of virus; year and location of the outbreak; place of HW exposure/employment; total numbers of persons who were exposed to and infected with the causal viruses; total number of persons who died from EVD/MVD; total numbers of HWs exposed to and infected with the causal viruses; total number of HWs who died from EVD/MVD; specific occupations (eg, nurse or environmental services staff) and numbers, by HW occupation, exposed or infected; exposure risk situations; and any breaches of IPC practices. Data were checked for accuracy by a second reviewer. PRISMA (Preferred Reporting Items for Systematic Reviews and Meta-analyses) guidelines were adhered to during the search, review, data collection, and analysis. When necessary, authors were contacted to clarify information from the reviewed studies.

The percentage of infected HWs among infected patients was calculated by dividing the absolute number of HW infections reported by the total number of EVD or MVD cases in each study/report. The EVD or MVD rate among HWs was calculated by dividing the number of HW infections reported by the number of HWs documented as having been exposed to EVD or MVD. The definition of an “exposed” HW relied on definitions used by the studies/reports and could be based on a HW’s self-report of exposure or the authors’ assumption that a HW was at risk for exposure, as reported in nationwide population-based studies. Similarly, case definitions varied according to the study/report, including whether probable and/or suspected infections were counted as cases. Owing to heterogeneity in the study designs/definitions, it was considered statistically inappropriate to perform a meta-analysis.

## RESULTS

Our search yielded 2983 records after removal of duplicates. After screening by title and abstract, 447 were selected for full-text review. Ninety-four articles were included in the final data set (Supplementary Figure 1). These included information about HW infections from 22 outbreaks (MVD, 9; EVD, 13), which occurred between 1967 and 2017 and affected 17 countries (Table 1).

**Table 1.** Health Worker (HW) Infections, by Year and Country Location

Country (Additional Geographic Descriptors)	Year	Virus (Species)	Patients Infected, No.	HWs Infected, No.	HWs Infected as % of All Infections
Germany, former Yugoslavia [2]	1967	Marburg	31	5	16
South Africa [48, 49]	1975	Marburg	3	1	33
Sudan [6]	1976	Ebola (S)	284	75	26
Zaire [5]	1976	Ebola (Z)	318	15	5
Zaire (Tandala) [47]	1972–1978	Ebola (Z)	6	1	17
Sudan [10]	1979	Ebola (S)	34	2	
Kenya [50]	1980	Marburg	2	1	50
Democratic Republic of the Congo [7, 9, 51–53, 56, 100]	1995	Ebola (Z)	315	80 [52] to 90 [97]	25 [52] to 32 [97]
Democratic Republic of the Congo [45, 54, 58]	1994	Marburg	20	3	15 [45]
	1997	Marburg	5	1	20 [45]
	1998–2000	Marburg	154	7	5 [45]
Uganda [28, 29, 39, 55, 56, 101]	2000	Ebola (S)	425	31	7 [29]
Republic of the Congo, Gabon [102, 103]	2001–2002	Ebola (Z)	124	2	2 [100]
Republic of the Congo [56, 104, 105]	2003 (Jan–Apr)	Ebola (Z)	143	3	2 [101, 102]
Republic of the Congo [106]	2003 (Nov–Dec)	Ebola (Z)	35	1	3
Angola (Uige hospital)^a^ [22, 57, 59]	2005	Marburg	392^a^	NR	NR [22]
Uganda [30, 40, 60]	2007–2008	Ebola (B)	116	14	12 [60]
Democratic Republic of the Congo^a^ [23]	2012	Ebola (B)	11^a^	NR	NR
Uganda [46]	2012	Marburg	14	2	14
Democratic Republic of the Congo [27]	2014	Ebola (Z)	69	8	12
Uganda [83]	2017	Marburg	5	2	40
**2013–2016 Ebola virus disease West Africa outbreak (species: Z**)					
**Country**	**Year**	**Descriptors of Epidemiologic Clusters/Case Series**	**Patients With EVD, No.**	**HWs With EVD, No.**	**HWs infected as % of All Infections**
Guinea [8, 13, 24, 42, 64, 81, 107, 108]	2013–2016	Conakry, Mar–Apr 2014	37	14	38 [8]
		Conakry, Boffa, and Telimele, Feb–Aug 2014	193	27	14 [105]
		Conakry ETC, Mar–Aug 2014	90	17	19 [104]
		Conakry, Jan 2014–Mar 2015	566	78	14 [13]
		Nationwide, Jan–Dec 2014	2210	162	8 [24]
Liberia [13, 31–33, 43, 61, 62, 64, 65, 81]	2014–2016	Nationwide, Mar–Aug 2014	810	97	12 [65]
		Montserrado, Mar–Aug 2014	223	38^c^	17 [65]
		Montserrado Jan 2014–Mar 2015	2829	136	4.8 [13]
		Margibi, Jan 2014–Mar 2015	839	53	6.3 [13]
		Lofa, Mar–Sep 2014	619	22	4 [32, 65]
		St. Paul Bridge cluster, Jan–Feb 2015	22	1	4.6 [33]
Sierra Leone [11, 13, 25, 34–38, 44, 63, 64, 67, 69–74, 76, 77, 81, 84, 85, 109–113]	2014–2016	Kenema, May 2014–Jan 2015	600	92	15 [71]
		Kenema ETU, Jul 2014	109	11	10 [44]
		Kenema, Jan 2014–Mar 2015	537	80	15 [13]
		Nationwide, May–Oct 2014	3854	199	5 [25, 63]
		Kailahun ETC, Jun–Oct 2014	489	28	6 [34]
		Kailahun, Jun–Dec 2014	354	18	5 [70]
		Bombali government hospital cluster, Oct–Nov 2014	2	1	50 [11]
		Cluster from maternity clinic and ward of general hospital in Tonkolili, Oct–Nov 2014	7	1	14 [11]
Country	Year	Descriptors of Epidemiologic Clusters/Case Series	Patients With EVD, No.	HWs With EVD, No.	HWs infected as % of All Infections
		Pujehun, 2014–2015	49	3	6 [76]
		Western Area, 2014–2015	4955	179^c^	3.6 [109]
		Koinadugu, 2014–2015	142	3	2.1 [110]
**Countries with Ebola virus disease importation followed by local transmission**					
Country	Year	Country Where Index Patient Was Infected (Ebola Virus species)	Patients Infected, No.	HWs Infected, No.	HWs Infected as % of All Infections
England [17]	1976	Sudan (S)^b^	1	1	100
South Africa [18]	1996	Gabon (Z)	2	2	100
Nigeria [12, 41, 66, 79, 82]	2014	Liberia (Z)	20	11 [12, 79] or 13 [41, 82]	55 [12, 79] or 65 [41, 82]
Spain [19, 20]	2014	Sierra Leone (Z)	3	3	100 [20]
USA [21, 26, 68, 75, 78, 80]	2014	Liberia (Z)	3	2	67 [21, 26, 78]

Abbreviations: B, *Bundibugyo ebolavirus*; NR, not reported; S, *Sudan ebolavirus*; Z, *Zaire ebolavirus*.

^a^For these 2 outbreaks, only data on HW deaths as a proportion of all deaths were available. The proportion of HW deaths out of total deaths was 4.6% in 2005 Angola MVD outbreak [22] and 27% in the 2012 Democratic Republic of Congo EVD outbreak [23].

^b^Laboratory technician infected via a needlestick injury while processing human tissue from Sudan as part of an outbreak investigation.

^c^Data extrapolated from the percentage of HWs reported as infected; the number was not reported originally in the citation.

### Proportion of Infected HWs Among Infected Patients

The location, year, and percentage of total infections, by EVD or MVD status, occurring in HWs are shown in Table 1. The percentage of all infected patients who were HWs ranged from 2% to 100%. Data from the 2013–2016 West Africa EVD outbreak show a range of 2.1% to 100% (n = 25; Table 1), depending on the cluster, similar to the range reported in other EVD outbreaks (n = 13; 2%–100%). HWs comprised 5%–50% of all cases in MVD outbreaks (n = 8). Most reports where HWs composed ≥50% of total infections involved small outbreaks in countries (the United Kingdom [17], South Africa [18], Nigeria [12], Spain [19, 20], and the United States [21]) where the index patients or tissue sources were originally infected in another country with an ongoing EVD/MVD outbreak (Table 1). In particular, of the 3 outbreaks where 100% of infected patients were HWs, only the outbreak in the United Kingdom in 1976 involved a laboratory technician; the outbreaks in Spain and South Africa affected other types of clinical staff (Table 1). Two outbreaks only had data on the proportion of HW deaths among the total number of deaths (4.6% in the 2005 Angola MVD outbreak [22] and 27% in the 2012 Democratic Republic of the Congo EVD outbreak [23]).

### Proportion of Infected HWs in the Exposed HW Population, Compared With the Proportion of Cases in the General Population

Data for the calculation of the EVD or MVD rate in the HW population were available in 21 reports, which reported on subsets of 6 different EVD and MVD outbreaks. In reports related to the recent West Africa outbreak (n = 15), the percentage of exposed HWs who developed EVD ranged from 0.6% to 92% (Table 2). In reports from earlier EVD/MVD outbreaks (n = 6), the percentage of exposed HWs who subsequently developed infection ranged from 12.5% to 76% (n = 3) for EVD and from 1% to 10% (n = 3) for MVD.

**Table 2.** Ebola Virus Disease and Marburg Virus Disease Rates in Health Workers (HWs) Exposed to Infected Patients

Country (Cluster)	Year	Virus	HW Infections as % of All Infections	HWs Exposed, No.	HWs Infected, No.	Exposed HWs Infected, %
South Africa [48]	1975	Marburg	33	35	1	3
South Africa [49]	1975	Marburg	33	100	1	1
Sudan (Maridi) [6]	1976	Ebola	28	230	72	31
Zaire (Yambuku Mission Hospital) [5]	1976	Ebola	5	17	13	76
Zaire (Ngaliema) [5]	1976	Ebola	5	16	2	12.5
Democratic Republic of the Congo (Kiwit General Hospital) [53]	1995	Ebola	25	427	37	9
Democratic Republic of the Congo [58]	1998–2000	Marburg	5	63	6	10
Sierra Leone (nationwide) [25]	2014	Ebola	5	2402	199	8
Sierra Leone (nationwide) [38]	2014	Ebola	NR	2435	293	12
Sierra Leone (Kenema Hospital ETU) [36]	2014	Ebola	5	27	24	89
Sierra Leone (Kenema Hospital ETU) [37]	2014	Ebola	NR	26	24	92
Sierra Leone (Kenema Hospital ETU) [71]	2014	Ebola	15	62	18	29
Sierra Leone (Kenema Hospital general wards) [71]	2014	Ebola	15	83	48	58
Sierra Leone (Kenema Hospital, all staff/volunteers) [71]	2014	Ebola	15	472	66	14
Sierra Leone (Kenema Hospital ETU, July 2014) [44]	2014	Ebola	10	45	11	24
Sierra Leone (Bombali district government hospital) [11]	2014	Ebola	50	39	1	2.6
Sierra Leone (maternity clinic and ward of general hospital in Tonkolili district) [11]	2014	Ebola	14	28	1	3.6
Guinea (nationwide) [24]	2014	Ebola	7.9	11529	162	1.4
Liberia (St. Paul Bridge Cluster) [33]	2015	Ebola	4.6	166	1	0.60
Spain [20]	2014	Ebola	100	117	1	0.85
USA [21, 26]	2014	Ebola	66	149	2	1.3

Abbreviations: ETC, Ebola treatment center; ETU, Ebola treatment unit; NR, not reported.

Six papers related to the 2013–2016 West Africa outbreak included data that could be used to compare an EVD infection rate (none were available for MVD) for HWs to that for non-HWs (Table 3). Two were population-based studies that calculated nationwide incidence rates for EVD in HWs versus the general population in Guinea [24] and Sierra Leone [25] during the 2013–2016 outbreak. In Guinea, HWs had a 47-fold increased risk for EVD as compared to the general population. In Sierra Leone, HWs had a 100-fold increased risk as compared to the general population. A third population-based report from the WHO [13] reported EVD infection rates for 3 subcategories of HWs only (physicians, nursing staff, and laboratory technicians) and calculated a 21–32-fold increased risk of infection in these HWs as compared to the risk for the general population.

**Table 3.** Ebola Virus Disease Rate in Health Workers Compared to Non–Health Workers (HWs) Exposed to Patients With Ebola

Country (Cluster)	Year	HWs Exposed, No.	% of Exposed HWs Infected	Non-HWs Exposed, No.	% of Exposed Non-HWs Infected
USA (Dallas, TX) [21, 26]	2014	149	1.3	30	0
Sierra Leone (Tonkolili maternity clinic and ward) [11]	2014	28	3.6	18	28
Sierra Leone [25]	2014	2402	8	3.49 million	0.08
Guinea [24]	2014	2210	1.4	6.15 million	0.03
Guinea, Liberia, and Sierra Leone [13]	2014–2015	General population infection rate, 0.14%; physician infection rate, 2.95%; registered nurse infection rate, 4.37%; laboratory technicians infection rate, 4.04%			

A further 3 studies provided data on infection rates in HWs who contact with index cases as compared to non-HWs with such contact. Of these, 2 reported no EVD infections in 20 non-HWs who had contact with the 3 EVD HW cases diagnosed in the US outbreak, compared with 2 infections among 149 HWs who had contact with these cases [21, 26]. One report tracked the transmission chain of nosocomial EVD spread in a maternity ward in Sierra Leone in October 2014 and found a higher infection rate in non-HW contacts (28%), compared with HW contacts (3.6%) [11].

### Most-Affected HW Occupations

**Figure 1.**
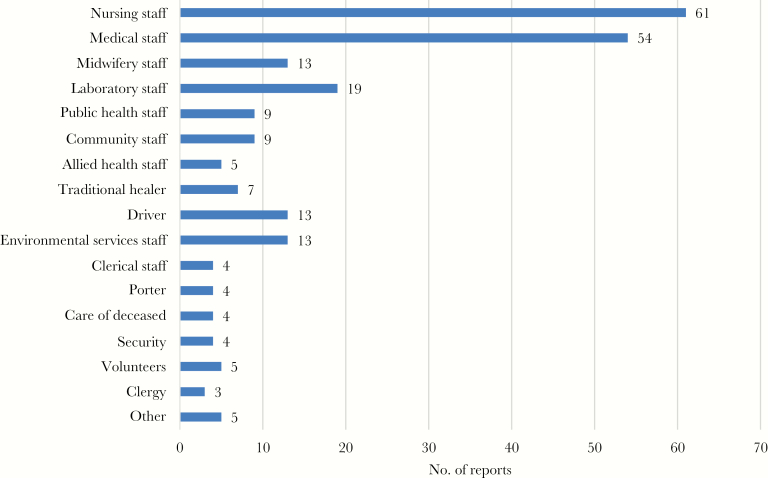
Most frequently exposed health worker occupations. The *x*-axis denotes the number of reports documenting exposure and/or infection in the specified occupation.

Figure 1 shows the HW occupations most exposed to EVD/MVD. Nursing staff were the most frequently identified (61 of 67 reports [91%]), followed by medical staff (54 of 67 [81%]) and laboratory staff (19 of 67 [28%]). Additional occupations identified were medical auxiliaries, students, pharmacists, phlebotomists, radiographers, counselors, transporters, burial teams, a prisoner asked to provide care in the hospital for another prisoner, and a construction worker on a building site at a healthcare facility. Supplementary Table 3 lists the occupations and titles identified as HWs.

### HW Mortality Rates

Twenty articles reported HW deaths resulting in case-fatality rates (CFRs) of 50% or greater due to EVD [5, 6, 8, 10, 11, 13, 18, 24, 27–38], including reports from the 2013–2016 West Africa outbreak [8, 11, 13, 24, 31–38] and from the 2014 Sudan outbreak, in which the CFR was 100% [27]). In 8 reports, the HW CFR from EVD ranged between 12% and 49% [12, 25, 39–44]. Among outbreaks of MVD with HW deaths, the HW CFR ranged from 50% to 100% [45, 46]. In some smaller outbreaks with limited local transmission of EVD or MVD, the reported HW CFR was 0% [2, 17, 21, 26, 47–50].

### Most-Frequent Exposure Risks

**Figure 2.**
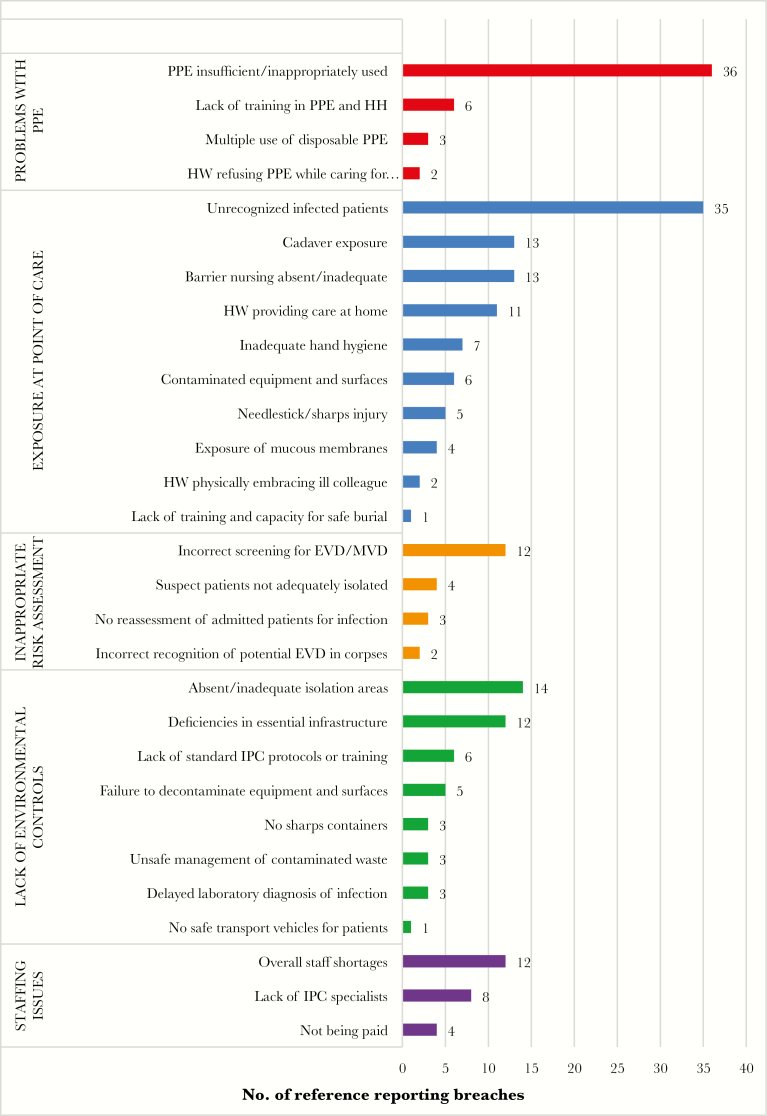
Factors leading to Ebola virus disease (EVD) and Marburg virus disease (MVD) exposure and infection in health workers (HWs). Abbreviations: HH, hand hygiene; IPC, infection prevention and control; PPE, personal protective equipment.

Risk situations and factors contributing to EVD/MVD HW exposure and infection were identified in 69 articles [5–7, 9–12, 17, 18, 21, 22, 24–28, 30–33, 35–44, 47, 48, 50–86] (Figure 2). Among the 5 major categories identified, “insufficient/incorrect use of personal protective equipment [PPE]” was the most frequently cited exposure risk. In many situations, deficiencies in PPE use arose from the lack of availability of appropriate equipment and/or the lack of training in PPE use during patient care, patient transport, and cleaning and environmental disinfection activities [5, 6, 9–11, 18, 22, 24–26, 28, 32, 36, 38–40, 42, 43, 50–54, 56, 58, 61–65, 69–74, 78–80, 85, 86]. Less commonly, HWs were observed to engage in behavior such as rubbing the eyes [52], smoking [9, 39], and using a mobile telephone [39], thus risking exposure to mucus membranes. One study found that the most frequent type of exposure incident (63 of 77 exposures among 57 HWs) was to skin on the face (including mucosa), because goggles/respirator masks did not stay correctly in place during patient care [42]. In one outbreak, HWs refused to wear PPE, to support the morale of infected coworkers [58].

Exposure at the point of care was the second most frequently cited exposure risk category in many reports, particularly to patients with unrecognized EVD/MVD [6, 9–12, 18, 21, 24, 26–28, 31–33, 35, 37, 38, 41, 43, 48, 50, 52, 57, 58, 60, 62, 64, 66, 67, 69, 72, 75, 82, 83, 85, 86] and to cadavers during unsafe burial practices [7, 22, 30, 39, 40, 48, 50, 51, 57, 59, 60, 65, 81]. Inadequate hand hygiene was a frequent factor leading to exposure at the point of care [9, 22, 28, 38, 40, 53, 62]. The third category of risk was inappropriate risk assessment, including lack of recognition of potential EVD in corpses [25, 52]. The fourth category related to a lack of environmental/engineering controls, including the absence of functional isolation wards or segregation [5, 10, 22, 25, 36, 39, 43, 48, 50–52, 58, 59, 62, 64, 65, 68, 74, 77, 86] and a lack of standard operating procedures to reduce the infection risk [25, 38, 68, 70, 75, 80, 86]. Several infrastructure deficiencies contributing to exposure risk were included in this category, such as a lack of electricity or running water; a lack of sharps disposal boxes; shortages of soap, chlorine, and other disinfection supplies; and inadequate/absent waste disposal methods [7, 10, 22, 25, 40, 44, 51, 56, 62, 63, 68, 70, 71]. In some areas, there were delays in the laboratory diagnosis of EVD/MVD [25, 32, 63] and a lack of safe transportation vehicles [25].

The fifth category was related to shortages of human resources. In particular, a lack of IPC specialists and frontline healthcare staff, combined with delayed/unpredictable payment of salaries [25, 36, 38, 39, 50, 51, 61, 63, 67, 68, 70–74, 84, 85], were identified as sources of provider stress that could contribute to risk exposure. One report noted that 4 of 5 infected HWs worked commonly or exclusively at night, which was also a risk factor for HW stress/fatigue and reduced levels of supervision [43].

In 2 clusters of infection, in the United States and Spain, where community exposure to EVD/MVD was not a factor, infected HWs did not report exposure due to specific IPC breaches during care [19–21]. One report from the Spain cluster proposed that the infected HW was “likely exposed to fomites” [20] during her work, although a specific incident related to fomite exposure was not identified. In the US cluster, one of the infected HWs reported after recovery that there were no standard IPC protocols in place for EVD at the hospital where she worked [80].

## DISCUSSION

To our knowledge, this is the first extensive systematic review investigating EVD and MVD in HWs and exploring the risk situations and factors leading to exposure in this population. We identified published reports from 74% of known EVD outbreaks and 70% of known MVD outbreaks [87, 88]. HW infections as a proportion of all cases in an EVD or MVD cluster or outbreak ranged from 2% to 100% (Table 1). Clusters with the highest proportion (ie, >50%) of EVD or MVD cases occurring in HWs were usually smaller outbreaks in countries where EVD/MVD was not circulating in the local population but introduced by an isolated traveler/individual. In areas with endemic or locally circulating EVD/MVD in the recent West Africa outbreak, the proportion of infected HWs ranged from 2.1% to 50%, similar to findings in earlier outbreaks (range, 2%–50%), contrasting with an overall figure of 3.9% reported by the WHO for 2014 to March 2015 [13]. The higher proportion of HWs cases in many of the reports included in our review is likely because many of the included clusters were from an early stage in the West Africa outbreak. At that time, there would have been less awareness among HWs of circulating EVD and the precautions necessary to prevent infection, as well as lower stocks of appropriate PPE and limited numbers of Ebola treatment centers and trained staff relative to the size of the outbreak (Table 1).

Data were limited on the proportion of HWs who became infected after EVD or MVD exposure (Table 2). Available data highlight great disparities between HW infection rates in countries where EVD and MVD are likely endemic in animal reservoirs (range, 3%–92%), compared with countries with smaller infection clusters due to importation (range, 0.85%–1.3%; n = 3 studies). Only 6 studies, all from the 2013–2016 EVD outbreak, compared infection rates in exposed HWs to rates in the general population/non-HWs, presumably because of the difficulty of assessing exposure in the community setting (Table 3). Three were population-based studies, which identified a 21–100-fold increase in the EVD rate in HWs, compared with that in the general population (Table 3 [13]). Only 1 paper, which tracked nosocomial EVD spread in a maternity ward, found a higher rate of infection in non-HW contacts versus HWs [11]. This may reflect both higher risk exposures in the non-HW contacts (peripartum women and newborns accounted for half of all infections), as well as an increased awareness of EVD and the use of PPE by HWs at the time of the study.

Mortality data were reported in a low number of included papers, and CFR among HWs varied significantly. In general, rates were >50% in both historical and recent outbreaks of EVD and MVD, although a few reports with information on HW deaths had CFRs between 10% and 40%. Overall, these findings are consistent with the results of 2 other systematic reviews/meta-analyses identified in our search, both with a focus on the 2013–2016 West Africa outbreak and reporting HW CFRs of ≥45% for affected countries [89, 90]. A recent study of 27 patients (22 HWs) with EVD treated in Europe and the United States reported 5 deaths, for a CFR of 18.5% [91], highlighting that while mortality may be lower in high-resource settings owing to timelier and/or more-appropriate treatment, a significant proportion of infections still results in death, including HW deaths.

Among HWs, nursing was the occupation most frequently mentioned as being exposed to EVD/MVD, consistent with data showing that infections involving nurses composed over half of all HW infections in the recent outbreak [13]. However, healthcare delivery has become increasingly complex by involving workers from many different occupations. Even with our wide search string, certain occupations associated with occupational EVD/MVD exposure were identified only during the data-extraction process, and future IPC education efforts should also take this into consideration.

Both the earliest documented outbreak of EVD, in 1976, and the recent 2013–2016 outbreak reported high infection rates of exposed HWs [5, 36]. This is likely associated with infection control deficiencies that were present in both earlier outbreaks and the recent outbreak, including a lack of PPE and environmental/engineering controls, lack of or inefficient triage and failure to recognize patients with EVD/MVD, and a shortage of human resources. Reports and surveys from the 1995 EVD outbreak in the Democratic Republic of the Congo identified nonfunctional isolation wards for suspected EVD cases, a lack of water and electricity, no waste disposal system, no PPE for medical staff, staff shortages, and inconsistent hand hygiene practices [51, 53]. Almost 2 decades later, similar deficiencies were reported in Sierra Leone and Liberia during the 2013–2016 outbreak [25, 33, 62]. The persistence of similar deficiencies through decades of outbreaks, combined with the continued high HW infection rate, emphasizes the need to improve the long-standing lack of IPC infrastructures and supplies and the poor adherence to standard precautions and occupational health and safety measures in all healthcare settings. This is also clearly confirmed by the Global Health Observatory, which reported joint external evaluations assessing country capacity to prevent, detect, and rapidly respond to public health risks. 2016 data from 64 countries showed that only 19% had demonstrated IPC capacity that accorded with international standards at the facility level; among low- and middle-income countries, this proportion was reduced to 2 of 38 countries [92]. In addition to improving current IPC practices and infrastructures, there is also urgent need for more-innovative PPE features and designs, particularly to address increased safety, usability, and comfort to best protect frontline HWs from filovirus transmission, especially in tropical climates. Based on international expert consensus, the WHO recently issued guidance on the characteristics of safer equipment, which will hopefully drive research and innovation [93].

Several studies compared the number of HW infections before and after the institution of IPC measures. In the Democratic Republic of the Congo 1995 EVD outbreak, the introduction of IPC measures at Kikwit General Hospital resulted in 1 HW infection as compared to 79 previously [51, 52]. Similarly, in the West Africa outbreak a reduction in the incidence of infection in HWs as a proportion of all cases (from 12% in July 2014 to 1% in February 2015) was observed, which may have been due to coordinated efforts by international and nongovernmental organizations to provide support and guidance leading to improved IPC practices [13]. Errors in the donning and doffing of PPE were recognized early on as contributing to the West Africa outbreak, leading to new interim guidance [14, 15]. A 2014 observational study in primary healthcare facilities in Kenema, Sierra Leone, found consistent glove reuse and poor hand hygiene. Donning and doffing in the correct order occurred in only 3% of observations. These factors improved significantly after appropriate training [94]. To lower infection rates even further, facilities must continue to educate and enforce the most up-to-date IPC guidelines and introduce systems for managing occupational health and safety, including work organization.

Working with patients who have unrecognized EVD/MVD was the second most commonly cited exposure risk mentioned in both earlier outbreaks and the recent outbreak. However, the presenting symptoms of EVD/MVD are also common to many other endemic illnesses that are far more frequent and do not necessarily require the same strict IPC measures. During outbreaks, exposure to unrecognized patients has been reduced by the use of triage tools, isolation of suspect cases, use of standard precautions and barrier nursing techniques, and improvement in laboratory infrastructures to reduce the time to diagnosis [51, 52], such as the introduction of new point-of-care tests for EVD that can be run quickly at health centers lacking laboratory facilities [95].

Phase 3 trials of the recombinant vesicular stomatitis virus Zaire Ebola virus vaccine have shown promising results as another method of reducing the infection risk in HWs who might be exposed during the initial triage and evaluation of patients [96]. However, data are still insufficient to establish whether the vaccine confers long-term protection, and preclinical studies in nonhuman primates suggest that the vaccine may not confer complete cross-protection against MVD and other EVD species known to be pathogenic in humans [97]. Until vaccination is demonstrated to confer long-term immunity against all species of EVD and MVD, the continued and appropriate use of IPC methods will remain crucial for protecting frontline HWs and preventing nosocomial spread of infection and amplified transmission out into the community.

The very high rate of EVD and MVD infections among HWs as compared to the general population indicates that all such infections should be considered as occupational diseases when they occur among HWs and other workers at high risk of exposure. The list of occupational diseases from the International Labour Organization (ILO) includes diseases caused by biological agents at work “where a direct link is established scientifically, or determined by methods appropriate to national conditions and practice, between the exposure to these biological agents arising from work activities and the disease(s) contracted by the worker (p. 3)” [98]. Such cases should be properly investigated to rule out nonoccupational exposure and notified as occupational diseases to the authority responsible for employment injury benefits. The WHO and ILO recommend that HWs with EVD and MVD resulting from work activities should have the right to compensation, as well as free rehabilitation and access to curative services [99].

Our study has some limitations. Notably, the heterogeneous nature of the retrieved publications limited the use of a more sophisticated analysis by pooling data. Several papers mentioned certain occupations as separate from HWs, even though these met our definition of “HW.” Occasional discrepancies were noted in numbers in published reports as compared to data from government/nongovernmental organizations [27, 45–47, 52, 53, 60, 71] and sometimes within reports related to the same cluster/outbreak [12, 41, 79, 82]. This may have been due to several factors, such as differences in case definitions and disparate definitions of both HWs and exposure between studies, resulting in different numbers of HWs reported as exposed or infected within the same outbreak [12, 41, 48, 49, 79, 82], and incomplete reporting to national databases [65].

To conclude, high HW infection rates and similar exposure risk factors in both past and recent EVD and MVD outbreaks highlight the need to urgently strengthen IPC program implementation at the facility level to ensure patient and HW safety in everyday care service delivery and in the event of an outbreak. Our data also represent a useful addition to inform models designed to estimate the impact of various prevention strategies and to emphasize that HWs also risk their lives for the patients under their care.

## Supplementary Material

Supplementary Table 1

Supplementary Table 2

Supplementary Table 3

Supplementary Figure 1

## Notes

***Acknowledgments.*** We thank Tomas John Allen and Jose Luis Garnica Carreno, for their assistance in creating the search strategy and obtaining references; Sergey Romualdovich Eremin, for his assistance in translating Russian texts; Maki Kajiwara and Hiroki Saito, for their assistance in translating Japanese texts; and Rosemary Sudan, for editing assistance.

B. A. had final responsibility for the decision to submit the manuscript for publication.

***Disclaimer.*** The World Health Organization takes no responsibility for the information provided or the views expressed in this article.

***Financial support.*** This work was supported by the World Health Organization.

***Potential conflicts of interest.*** All authors: No reported conflicts.

Presented in part: Maryland American College of Physicians Mulholland-Mohler Residents Meeting, Baltimore, Maryland, 26 May 2016.
