# Supplementary material for: Infection Rates and Risk Factors for Infection Among Health Workers During Ebola and Marburg Virus Outbreaks: A Systematic Review
Source: J Infect Dis. 2018 Sep 7;218(Suppl 5):S679–89. doi: 10.1093/infdis/jiy435 (PMC6249600; doi:10.1093/infdis/jiy435)
Supplement: Supplementary Table 1 [file jiy435_suppl_jiy435_suppl_supplementary_table_1.docx]

**Supplementary Table 1. Search Terms Used for the Systematic Review - PubMed.**

| 1 | "Health Personnel"[MeSH] OR "health personnel"[all fields] OR "healthcare personnel"[all fields] OR "health care personnel"[all fields] OR “health worker”[all fields] OR “health workers”[all fields] OR “healthcare worker”[all fields] OR “healthcare workers”[all fields] OR “health care worker”[all fields] OR “health care workers”[all fields] OR “healthcare provider”[all fields] OR “healthcare providers”[all fields] OR “health care provider”[all fields] OR “health care providers”[all fields] OR “health practitioner”[all fields] OR “health practitioners”[all fields] OR "healthcare practitioner"[all fields] OR “health care practitioner”[all fields] OR "healthcare practitioners"[all fields] OR “health care practitioners”[all fields] OR “health employee”[all fields] OR “health employees”[all fields] OR "medical staff"[all fields] OR doctor[all fields] OR doctors[all fields] OR physician*[all fields] OR ("allied health"[all fields] AND ("staff"[all fields] OR personnel[all fields])) OR paramedic[all fields] OR paramedics[all fields] OR "nursing staff" [all fields] OR nurse[all fields] OR nurses[all fields] OR "hospital personnel"[all fields] OR "hospital staff" [all fields] OR “hospital worker”[all fields] OR “hospital workers”[all fields] OR (“burial”[MeSH] AND (staff OR personnel OR workers)) OR “traditional medicine”[all fields] OR “traditional healer”[all fields] OR “traditional healers”[all fields] OR “burial worker”[all fields] OR “burial workers”[all fields] OR “funeral worker”[all fields] OR “funeral workers”[all fields] OR “taxi driver”[all fields] OR “taxi drivers”[all fields] OR “security worker”[all fields] OR “security workers”[all fields] OR “security”[all fields] AND (staff OR personnel OR workers)) OR “volunteers”[all fields] OR “community worker”[all fields] OR “community workers”[all fields] OR (“cleaner”[all fields] AND (staff OR personnel OR workers)) OR “janitors”[all fields] OR “custodians”[all fields] OR “laboratory worker”[all fields] OR “laboratory workers”[all fields] OR (“laboratory”[all fields] AND (staff OR personnel OR workers)) OR “traditional healers”[all fields] OR “traditional midwives”[all fields] OR midwi*[all fields] OR “religious leaders”[all fields] OR “contact tracer”[all fields] OR “contract tracers”[all fields] OR “public health worker”[all fields] OR “public health workers”[all fields] OR “community worker”[all fields] OR “community workers”[all fields] OR “point of entry worker”[all fields] OR pilot*[all fields] OR “flight attendant”[all fields] OR “ship worker”[all fields] OR “soldiers”[all fields] OR (“military”[all fields] AND (staff OR personnel OR workers)) OR “community health centers”[MeSH] OR “community health services”[MeSH] OR “community health nursing”[MeSH] OR “community health workers”[MeSH] OR “volunteers”[MeSH] OR “social work”[MeSH] OR “Infectious disease transmission, patient-to-professional”[MeSH] OR “military personnel”[MeSH] OR “laboratory personnel”[MeSH] OR “medical laboratory personnel”[MeSH] OR “medicine, traditional”[MeSH] OR “midwifery”[MeSH] OR “air travel”[MeSH] OR “missionaries”[MeSH] OR “medical missions, official” [MeSH] OR hygienist*[all fields] OR “water and sanitation workers”[all fields] OR “watsan worker”[all fields] OR “watsan workers”[all fields] OR (administrat*[all fields] AND (“staff”[all fields] OR personnel[all fields])) OR (“communication”[all fields] AND (“staff”[all fields] OR personnel[all fields])) OR (“sanitation”[all fields] AND (staff OR personnel OR workers)) OR (“waste management”[all fields] AND (staff OR personnel OR workers)) OR “community liaison”[all fields] OR “community liaisons”[all fields] OR "Students, Health Occupations"[Mesh] OR “health auxiliary”[all fields] OR “health care manpower”[all fields] OR “medical personnel”[all fields] OR “nursing home personnel”[all fields] OR “paramedical personnel”[all fields] OR “General Practitioners”[all fields] OR “General Practitioner”[all fields] OR Caregiver*[all fields] OR “Medical students”[all fields] OR “Nursing students”[all fields] OR (health*[all fields] AND worker*[all fields]) OR “Community Health Workers”[all fields] OR “Community Health Worker”[all fields] OR “Dental Staff”[all fields] OR dentist[all fields] OR dentists [all fields] |
| --- | --- |
| 2 | "Hemorrhagic Fever, Ebola"[Mesh] OR "Ebolavirus"[Mesh] OR "Ebola Vaccines"[Mesh] OR Ebola [All]  OR EVB [TIAB] OR "Marburg Virus Disease"[Mesh] OR "Marburgvirus"[Mesh] OR Marburg[TW]   OR marburgvirus* [all] OR (Marburg* [tw] AND (syndrome*[tw]  OR fever*[tw]  OR haemorrhag*[tw]  OR hemorrhag*[tw]  OR disease*[tw]  OR virus*[tw])) |
