# Supplementary material for: Infection Rates and Risk Factors for Infection Among Health Workers During Ebola and Marburg Virus Outbreaks: A Systematic Review
Source: J Infect Dis. 2018 Sep 7;218(Suppl 5):S679–89. doi: 10.1093/infdis/jiy435 (PMC6249600; doi:10.1093/infdis/jiy435)
Supplement: Supplementary Table 2 [file jiy435_suppl_jiy435_suppl_supplementary_table_2.docx]

**Supplementary Table 2. Search Terms Used for the Systematic Review - EMBASE.**

| 1 | 'health personnel'/exp OR 'health personnel':ti,ab,de OR ‘healthcare personnel’:ti,ab,de OR ‘health care personnel’:ti,ab,de OR ‘health worker’:ti,ab,de OR ‘health workers’:ti,ab,de OR ‘healthcare worker’:ti,ab,de OR ‘healthcare workers’:ti,ab,de OR ‘health care worker’:ti,ab,de OR ‘health care workers’:ti,ab,de OR ‘healthcare provider’:ti,ab,de OR ‘healthcare providers’:ti,ab,de OR ‘health care provider’:ti,ab,de OR ‘health care providers’:ti,ab,de OR ‘health practitioner’:ti,ab,de OR ‘health practitioners’:ti,ab,de OR ‘healthcare practitioner’:ti,ab,de OR ‘health care practitioner’:ti,ab,de OR ‘healthcare practitioners’:ti,ab,de OR ‘health care practitioners’:ti,ab,de OR ‘health employee’:ti,ab,de OR ‘health employees’:ti,ab,de OR ‘medical staff’:ti,ab,de OR doctor:ti,ab,de OR doctors:ti,ab,de OR physician*:ti,ab,de OR ‘General Practitioners’:ti,ab,de OR ‘General Practitioner’:ti,ab,de OR (‘allied health’:ti,ab,de AND (‘staff’:ti,ab,de OR personnel:ti,ab,de)) OR paramedic:ti,ab,de OR paramedics:ti,ab,de OR ‘nursing staff’:ti,ab,de OR nurse:ti,ab,de OR nurses:ti,ab,de OR ‘hospital personnel’:ti,ab,de OR ‘hospital staff’:ti,ab,de OR ‘hospital worker’:ti,ab,de OR ‘hospital workers’:ti,ab,de OR ‘burial worker’:ti,ab,de OR ‘burial workers’:ti,ab,de OR ‘funeral worker’:ti,ab,de OR ‘funeral workers’:ti,ab,de OR ‘funeral staff’:ti,ab,de OR ‘funeral personnel’:ti,ab,de OR ‘burial staff’:ti,ab,de OR ‘burial personnel’:ti,ab,de OR ‘traditional healer’/exp OR ‘traditional healer’:ti,ab,de OR ‘traditional healers’:ti,ab,de OR ‘taxi driver’:ti,ab,de OR ‘taxi drivers’:ti,ab,de OR ‘ambulance driver’:ti,ab,de OR ‘taxi driver’/exp OR ‘emergency health service’/exp OR (security NEXT/1 (staff OR personnel OR workers OR worker)) OR 'security staff'/exp OR (‘non profit organization’ NEXT/1 (staff OR personnel OR workers OR worker OR volunteer OR volunteers)) OR ‘community worker’:ti,ab,de OR ‘community workers’:ti,ab,de OR ‘relief work’/exp OR ‘voluntary worker’ OR (clean* NEXT/1 (staff OR personnel OR workers)) OR ‘janitors’:ti,ab,de OR ‘custodians’:ti,ab,de OR ‘hospital cleaning worker’/exp OR (laboratory NEXT/1 (staff OR personnel OR worker OR workers)) OR midwi*:ti,ab,de OR 'religious personnel'/exp OR 'religious personnel':de,ab,ti OR 'religious leaders':de,ab,ti OR ‘community health centers’:ti,ab,de OR ‘community health services’:ti,ab,de OR ‘community health nursing’ OR (health* NEXT/1 worker*):de,ab,ti OR ‘community health worker’ OR ‘public health worker’:ti,ab,de OR ‘public health workers’:ti,ab,de OR ‘contact tracer’ OR ‘contract tracers’ OR 'social worker' OR 'social workers' OR 'air travel' OR 'airplane crew' OR 'aeroplane pilot' OR 'airplane pilot' OR 'flight attendant' OR 'airplane pilots' OR 'flight attendants' OR 'aeroplane pilots' OR 'sailor'/exp OR (ship* NEXT/1 (staff OR personnel OR worker OR workers)) OR 'soldiers':de,ab,ti OR 'soldier':de,ab,ti OR (military NEXT/1 (staff OR personnel OR workers OR worker)):de,ab,ti OR ‘air force’/exp OR ‘navy’/exp OR ‘army’/exp OR ‘civil defense’/exp OR ‘military deployment’/exp OR ‘military service’/exp OR (sanitation NEXT/1 (staff OR personnel OR workers OR worker)) OR ('waste management' NEXT/1 (staff OR personnel OR workers OR worker)) OR ‘watsan’ OR (administrat* NEXT/1 (staff OR personnel OR worker OR workers)) OR 'nursing student':de,ab,ti OR 'medical student':de,ab,ti OR ‘caregiver’ OR 'dental staff':de,ab,ti OR dentist:de,ab,ti OR dentists:de,ab,ti OR 'dental assistant':de,ab,ti OR 'dental assistants':de,ab,ti |
| --- | --- |
| 2 | Ebola OR 'Marburg hemorrhagic fever'/exp OR (Marburg*:ti,ab,de AND (syndrome OR fever* OR haemorrhag* OR hemorrhag* OR disease* OR virus*):ti,ab,de) OR 'Marburg virus'/exp OR 'Ebolavirus'/exp OR 'Ebola hemorrhagic fever'/exp OR 'Ebola vaccine'/exp OR EVB:ti,ab |
