# Supplementary material for: Infection Rates and Risk Factors for Infection Among Health Workers During Ebola and Marburg Virus Outbreaks: A Systematic Review
Source: J Infect Dis. 2018 Sep 7;218(Suppl 5):S679–89. doi: 10.1093/infdis/jiy435 (PMC6249600; doi:10.1093/infdis/jiy435)
Supplement: Supplementary Table 3 [file jiy435_suppl_jiy435_suppl_supplementary_table_3.docx]

**Supplementary Table 3. Table Defining Health Worker Classifications**

| Classification | Mention by number of references | Additional notes on occupations within the category |
| --- | --- | --- |
| Nursing staff | 61 | includes nurses (47), nursing nuns (3), nursing assistants/aides (7), nursing students (4) |
| Medical staff | 54 | includes physicians (42), medical assistants (6), medical students (4), surgeon (1) anesthetist (1) |
| Midwifery staff | 13 | includes midwives (12), nurse midwives (1) |
| Laboratory staff | 19 |  |
| Public health staff | 9 | includes vaccinators/contact tracers (4), surveillance (2) and 3 unspecified |
| Community staff | 9 | includes 6 community health workers and 3 mother-and-child aides |
| Allied health staff | 5 | includes 4 pharmacists and 1 radiographer |
| Traditional healer | 7 |  |
| Driver | 13 |  |
| Cleaner | 13 |  |
| Clerical staff | 4 |  |
| Porter | 4 |  |
| Care of deceased | 4 | includes mortuary attendant (2) and burial team (2) |
| Security | 4 | includes security forces (2), army (1), police (1) |
| Volunteers | 5 |  |
| Clergy | 3 |  |
| Other | 5 | includes ward maid (1), admission staff (1), room attendant (1), construction worker (1), and prisoner (1) |
