# Supplementary figures and images for: Infection Rates and Risk Factors for Infection Among Health Workers During Ebola and Marburg Virus Outbreaks: A Systematic Review
Source: J Infect Dis. 2018 Sep 7;218(Suppl 5):S679–89. doi: 10.1093/infdis/jiy435 (PMC6249600; doi:10.1093/infdis/jiy435)

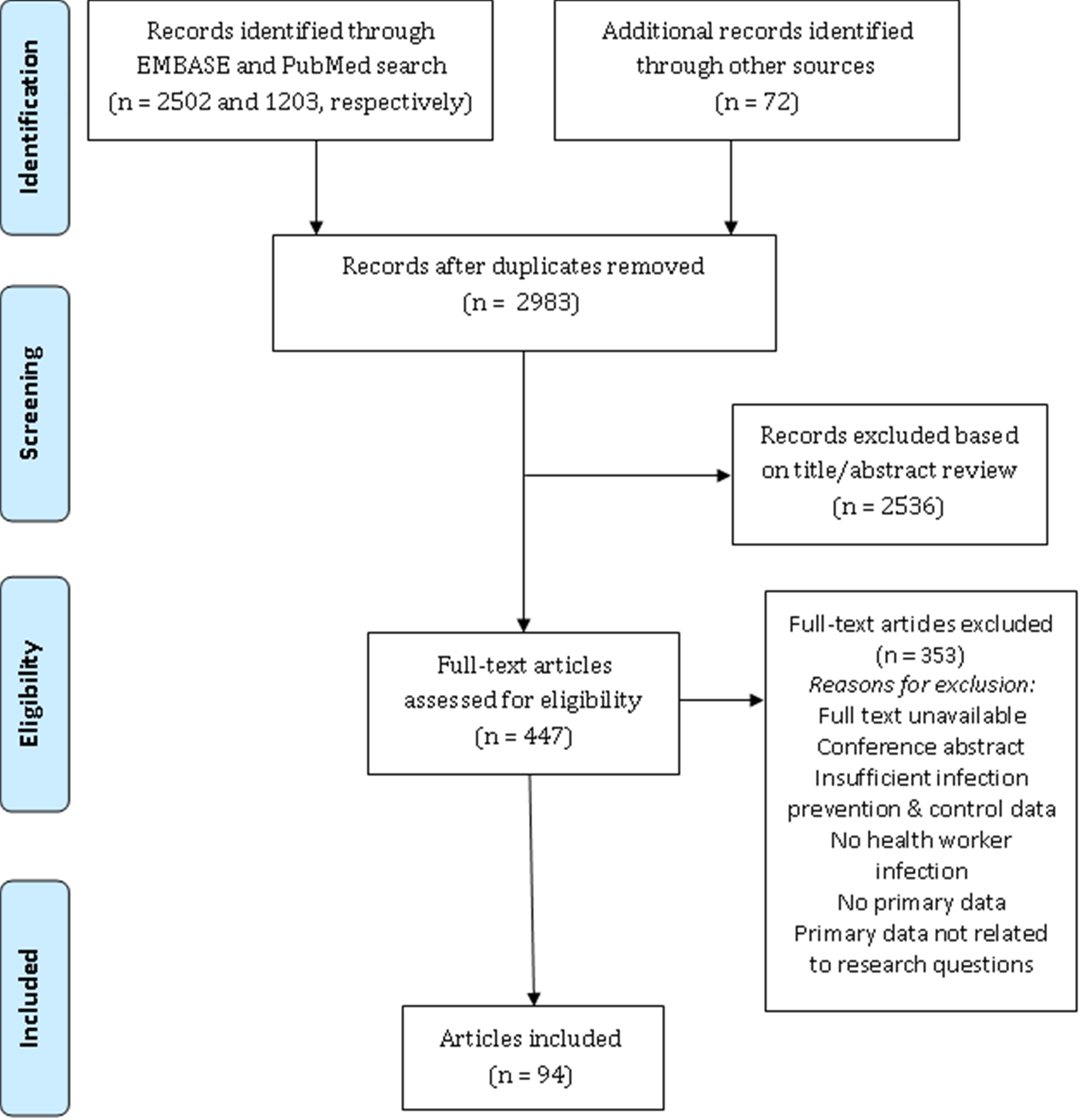

Supplement: Supplementary Figure 1 [file jiy435_suppl_supplementary_figure_1.png]
